# The Transcription Factor Foxc1 Promotes Osteogenesis by Directly Regulating Runx2 in Response of Intermittent Parathyroid Hormone (1–34) Treatment

**DOI:** 10.3389/fphar.2020.00592

**Published:** 2020-05-05

**Authors:** Ningjuan Ouyang, Hongliang Li, Minjiao Wang, Hongzhou Shen, Jiawen Si, Guofang Shen

**Affiliations:** ^1^Department of Orthodontics, Shanghai Ninth People’s Hospital, Shanghai Jiao Tong University School of Medicine, Shanghai Key Laboratory of Stomatology, Shanghai, China; ^2^Department of Oral and Cranio-maxillofacial Surgery, Shanghai Ninth People’s Hospital, Shanghai Jiao Tong University School of Medicine, Shanghai Key Laboratory of Stomatology, Shanghai, China

**Keywords:** parathyroid hormone, Foxc1, Runx2, osteogenic differentiation, bone regeneration

## Abstract

Parathyroid hormone (PTH) is crucial for bone remodeling. Intermittent PTH (1–34) administration stimulates osteogenesis and promotes bone formation; however, the possible targets and underlying mechanisms still remain unclear. In this study, functional links between PTH and Foxc1, a transcription factor reported to be predominant in skeletal development and formation, were indicated. We determined the impacts of Foxc1 on *in vitro* osteogenic differentiation and *in vivo* bone regeneration under intermittent PTH induction, and further explored its possible targets. We found that the expression level of Foxc1 was upregulated during osteogenic induction by intermittent PTH treatment, and the elevated expression of Foxc1 induced by PTH was inhibited by PTH1R silencing, while rescued by intermittent PTH supplement. By gain- and loss-of-function strategies targeting Foxc1 in MC3T3-E1 cells, we demonstrated that Foxc1 could promote *in vitro* osteogenic differentiation by intermittent PTH induction. Moreover, immunofluorescence analysis indicated the nuclear co-localization of Foxc1 with Runx2. Luciferase-reporter and chromatin immunoprecipitation analysis further confirmed that Foxc1 could bind to the P1 promoter region of Runx2 directly, which plays an indispensable part in osteogenic differentiation and bone mineralization. Meanwhile, we also revealed that Foxc1 could promote bone regeneration induced by intermittent PTH treatment *in vivo*. Taken together, this study revealed the role and mechanism of Foxc1 on *in vitro* osteogenic differentiation and *in vivo* bone regeneration in response of intermittent PTH treatment.

## Introduction

Parathyroid hormone (PTH) is released from the parathyroid gland responding to low serum calcium values and has significant impacts on bone metabolism (Delgado-Calle et al., 2017). As a major regulator of skeletal remodeling, PTH mediates both anabolic and catabolic processes of bone. PTH or its biological N-terminal fragment PTH (1–34) has been used as a therapy for osteoporosis for decades (Esen et al., 2015). Clinical researches indicated that intermittent PTH (1–34) administration could improve bone mass and decrease the risk of fracture in osteoporosis sufferers (Gardinier et al., 2015). Pharmacological daily injections of PTH results in a substantial rise in bone formation, attributing to the effects of PTH on promoting proliferation of pre-osteoblast cells, inhibiting apoptosis of osteoblasts, reactivating formation of lining cells to osteoblasts which synthesize matrix, or a mix of the above actions (Ben-Awadh et al., 2014). Even though the impacts of intermittent PTH treatment on bone have been well reported, the targets and the underlying mechanisms responsible for bone formation induced by intermittent PTH have not been fully elucidated yet.

Meanwhile, the regulation of osteogenesis usually involves the collaborative activities of several signaling proteins and transcription factors (Hopkins et al., 2016b). Foxc1, which belongs to the transcription factors family named as Forkhead box or FOX genes, has an essential impact on regulation of intramembranous and endochondral ossification in normal development and bone formation (Mya et al., 2018a). Foxc1 null embryos displayed bony fusion of maxilla and mandible, accompanied by defects in maxilla, mandible, and agenesis of the temporomandibular joint (Inman et al., 2013). Foxc1 knockout mice showed severe defect in vertebrae, axial skeleton, and skull. Notably, their calvarium, consisted of the parietal, frontal, and intraparietal bones, were completely absent(Motojima et al., 2016). Mesenchyme progenitor cells from Foxc1 mutant mice were unable to differentiate into chondrocytes and osteoblast cells. Meanwhile, knockdown of Foxc1 levels by siRNA in C2C12 cells inhibited the process of osteogenesis (Hopkins et al., 2016b). What is more, in our previous studies, we also found that Foxc1 expression gradually increased during osteogenesis in human amniotic epithelial cells, amniotic fluid derived mesenchymal stem cells, and bone marrow mesenchymal stem cells (Si et al., 2015).

Notably, numbers of molecules had been proved to be important in response of PTH on osteogenesis, including IGF, NFATc1, BMP4, Msx2, and so on (Nagel et al., 2015; Khan et al., 2016), and they were also proved to be the downstream molecules of Foxc1 during osteogenesis (Wang et al., 2016b; Hariri et al., 2018). In the meantime, we also found that the expression level of Foxc1 was upregulated during osteogenic induction by intermittent PTH treatment. These findings implied a possible relationship between Foxc1 and PTH in osteogenic differentiation. As yet, the specific function of Foxc1 in PTH-induced osteogenesis is still unknown.

Herein, we determined the effect of Foxc1 on *in vitro* osteogenic differentiation and *in vivo* bone regeneration under intermittent PTH treatment, and further explored its possible targets for better understanding of the molecular mechanism.

## Materials and Methods

### Cell Culture

Rat bone mesenchymal stem cells (rBMSCs) were isolated from rat tibias and femurs, and cultured as previously described (Lennon and Caplan, 2006). Cells were cultured in Dulbecco’s modified Eagle’s essential medium (DMEM, Gibco, USA) with 10% fetal bovine serum (FBS, Gibco, USA), 100 U/ml penicillin G, and 100 μg/ml streptomycin, in a humidified incubator at 37°C with 5% CO_2_. Mouse pre-osteoblastic MC3T3-E1 cells (ATCC, USA) were cultured in α-Minimum Essential Medium (α-MEM, HyClone, USA) with 10% FBS (HyClone, USA) and 1% penicillin G and streptomycin. To simulate intermittent exposure of PTH, 10 nM PTH (1–34) treatment was conducted for 6 h, followed by basic medium treatment in the next 42 h following a 48-h cycle (Sun et al., 2009).

### PTH1R Silencing

PTH1R small interfering RNAs (siRNA) were synthesized by Genomeditech (China) (sequences in **Table 1**). PTH1R siRNA (50 nM) was transfected into rBMSCs cells by Lipofectamine2000 according to the manufacturer’s protocol (Invitrogen, USA). Knockdown efficiency was measured by qPCR and Western blot after 48 h.

**Table 1:** siRNA sequences of PTH1R.

Primer	Sequence
PTH1R-F	GGCAGAUCCAGAUGCAUUATT
PTH1R-R	UAAUGCAUCUGGAUCUGCCTT

### Lentivirus (LV) and Adeno-Associated Virus (AAV) Preparation and Transfection

Lentiviruses for Foxc1 gene overexpression, and Foxc1 silencing were constructed. Briefly, the full‐length mouse Foxc1 gene, short hairpin RNAs targeting Foxc1 were encoded into different vectors obtained from Genomeditech (China) (Sequencing results in **Supplementary Data 1, 2**). The vectors and their packaging plasmids were transfected at the same time into 293T cells by Lipofectamine2000 (Invitrogen, USA). After changing the medium 8 h after transfection, the supernatant was collected, filtered, and went through ultracentrifugation 40 h later. 293 T cells were then transduced with serially diluted lentivirus. Four days after that, recombinant high‐titer lentiviral vectors with Foxc1 and shFoxc1 were collected.

Cells were seeded in 6‐well plates (1 × 10^5^ cells/well) and transduced with the control lentiviruses (LV‐vector or Lv-scramble), Foxc1‐overexpressing lentivirus (LV‐Foxc1) (primer sequences of expression plasmid in **Table 2**), and short hairpin Foxc1 lentivirus (LV‐shFoxc1–1, LV‐shFoxc1–2, LV‐shFoxc1–3) (primer sequences of interfering plasmids in **Table 3**). A 10-µg/ml polybrene was used for improvement of transduction efficiency.

**Table 2:** Primer sequences of expression plasmid.

Primer	Sequence (5′to 3′)
Foxc1-F (EcoRI)	CCGGAATTCATGCAGGCGCGCTACTCGG
Foxc1-R (BamHI)	CCGGGATCCTCAGAATTTGCTACAGTCATAGACGAAAGCC

**Table 3:** Target sequences of interfering plasmids of mouse Foxc1.

Plasmid	Target Sequence (5′to 3′)
shRNA1	GCACAACCTCTCGCTTAATGA
shRNA2	GCGGGAAATGTTCGAGTCTC
shRNA3	GAATGGGAATAGTAGCTGTCA

Meanwhile, Foxc1-ShRNA plasmid (primer sequences in **Table 4** and sequencing results in **Supplementary Data 3**) was introduced into 293 adeno-associated virus (AAV) cells to generate viruses (AAV-shFoxc1) for rat Foxc1 knockdown, and the control virus was packaged with the plasmid pAAV-ZsGreen-ShRNA (AAV-scramble). rBMSCs were transduced with AAV at a multiplicity of infection (MOI) of 50 and cultured for 2 days (Chen et al., 2018). For transplantation *in vivo*, the cells were allowed to recover for 24 h before harvesting.

**Table 4:** Target sequences of interfering plasmids of rat Foxc1.

Plasmid	Target Sequence (5′to 3′)
shRNA	GAATGGGAATAGTAGCTGTCA

### RT-qPCR

Total cellular RNA was isolated by a standard protocol with easy dilution kit from Takara (Japan). After reverse transcription reaction according to RT reagent kit also from Takara (Japan), qPCR was carried out with the following cycles: stage1: 95°C for 30 s; stage 2: 95°C for 5 s, 60°C for 30 s for 40 cycles. Sequences of the primers are shown in **Tables 5** and **6**.

**Table 5:** Real-Time PCR primer sequences for target genes (rBMSCs).

Gene(rat)	Sequence
GAPDH	Forward: 5′-CACCCGCGAGTACAACCTTC-3′
	Reverse: 5′-CCCATACCCACCATCACACC-3′
Foxc1	Forward: 5′-GCTACATCGCTCTTATCACCA-3′
	Reverse: 5′-GTTGTGCCGTATGCTGTTCT-3′
Pth1r	Forward: 5′-AGGTGGTTCCAGGGCACAA -3′
	Reverse: 5′-CAACTCTTCCTCTGTGAGGC -3′
Runx2	Forward: 5′-GCACAAACATGGCCAGATTCA-3′
	Reverse: 5′-AAGCCATGGTGCCCGTTAG-3′
OSX	Forward: 5′-AGGCACAAAGAAGCCATACG-3′
	Reverse: 5′-GGGAAGGGTGGGTAGTCATT-3′
OCN	Forward: 5′-GCTGCCCTAAAGCCAAACTCT-3′
	Reverse: 5′-AGAGGACAGGGAGGATCAAGTTC-3′

**Table 6:** Real-Time PCR primer sequences for target genes (MC3T3-E1).

Gene(mice)	Sequence
GAPDH	Forward: 5′-GACTTCAACAGCAACTCCCAC-3′
	Reverse: 5′-TCCACCACCCTGTTGCTGTA-3′
Foxc1	Forward: 5′-GAACTTCCACTCGGTGCG-3′
	Reverse: 5′-GCTACAGTCATAGACGAAAGCC-3′
Runx2	Forward: 5′-GCACAAACATGGCCAGATTCA-3′
	Reverse: 5′-AAGCCATGGTGCCCGTTAG-3′
OSX	Forward: 5′-AGGCACAAAGAAGCCATACG-3′
	Reverse: 5′-GGGAAGGGTGGGTAGTCATT-3′
OCN	Forward: 5′-GCTGCCCTAAAGCCAAACTCT-3′
	Reverse: 5′-AGAGGACAGGGAGGATCAAGTTC-3′

### Western Blot

Protein was extracted with RIPA Lysis Buffer at 4°C. After quantification, an equal volume of cell lysate was loaded and separated on a 10% SDS-PAGE gels and then transferred to polyvinylidene fluoride (PVDF) membranes (Millipore, USA). After blocking with 5% nonfat milk in PBS, the membrane was incubated with primary antibodies at 4°C overnight (anti-Foxc1 (1/1000; CST, USA), anti-Runx2 (1/2000; Abcam, USA), anti-Osx (1/800; Abcam, USA), anti-Ocn (1/1000; Abcam, USA), and anti-GAPDH (1/2000; CST, USA). The membrane was washed with Tris-buffered saline plus 0.1% Tween 20 (TBST), and incubated with fluorescent secondary antibodies (anti-rabbit IgG and anti-rat IgG, 1/10000; CST, USA). The blot was scanned by Odyssey Infrared Imaging System (USA).

### Alkaline Phosphatase Staining and Quantitative Analysis

For alkaline phosphatase (ALP) staining, cells were soaked in BCIP/NBT solution (Beyotime, China) in darkness, and purple-stained areas were considered positive. For level of ALP activity, the detection was performed following the manufacturer’s instructions (Beyotime, China). Briefly, 400-μl lysis buffer was added to the samples followed by a 4-h incubation at 37°C and a 30-min vibration at room temperature. After that, samples were mixed with p-Nitrophenyl phosphate (p-NPP) and substrate buffer, followed by a 10-min vibration and a 15-min incubation at 37°C. The enzyme activity was measured at 405 nm (BioTek, USA). The protein amount was obtained with the Bradford method in aliquots with the protein assay kit (Bio-Rad, USA), and absorbance was measured at 630 nm with protein concentration was being calculated according to a series of BSA standards (Sigma, USA). ALP activity was determined as absorbance at 405 nm per mg of total cellular proteins.

### Alizarin Red S Staining and Calcium Deposition Assay

After fixation and an additional washing, cells were soaked in 40 mM Alizarin red S (ARS) solution (pH 8.8) at 37°C for 30 min. On the other hand, the calcium deposition analysis was carried out following the manufacturer’s instructions (Sigma, USA). Briefly, a standard curve was prepared by Calcium Standard into a series of wells to give 0, 0.4, 0.8, 1.2, 1.6, 2.0 μg calcium per well. A 90-μl chromogenic reagent and a 60-μl calcium assay buffer were then added to each well containing standards, samples, or controls and mix gently followed by a 5-min incubation at room temperature. After that, the absorbance was measured at 595 nm, and the calcium concentration was calculated by the following equation: C = Sa/Sv (μg/μl). Sa is the calcium sample amount (in μg) obtained from standard curve. Sv is the sample volume (μl) added into the sample well.

### Cell Proliferation Assay

Cells were seeded into 96-well plates at the density of around 3,000 cells per well counted with cell counters under an inverted phase contrast microscope (Leica, Germany). After adherence, the cells were treated with 10-μl CCK-8 reagent (Dojindo Laboratories, Japan) for 2 h every day from 1st day to 7th day. The absorbance was detected at 405 nm.

### Immunofluorescence

Cells were fixed in 4% paraformaldehyde (PFA) for 15 min and then washed with PBS. After incubation in a 5% bovine serum albumin (BSA, Sigma, USA) containing 3‰ Triton X-100 for 1 h, cells were then incubated with the following primary antibodies dissolved in 5% BSA at 4°C overnight: anti-Foxc1 (1/200; CST, USA), anti-Runx2 (1/500; Abcam, USA). On the next day, cells were incubated with the following secondary antibodies for an hour: Alex Fluor 594 and Alex Fluor 488 (1/1000; Abcam). DAPI solution (Invitrogen, USA) was used for nuclear staining. Images were taken with a Canon fluorescence microscope (Japan).

### Luciferase Assay

Cells were seeded into a 24-well plate (at a density of around 4 × 10^4^ cells/well) 24 h before transfection. On the next day, cells were transduced with 50 ng of mouse Runx2 reporter construct (Genomeditech, China), along with 250 ng of overexpression Foxc1 vector or control vector. Luciferase-reporter assays were conducted following the manufacturer’s instructions (Promega, USA).

### Chromatin Immunoprecipitation

Chromatin immunoprecipitation (ChIP) assay was conducted following the manufacturer’s protocol (Millipore, USA). Cells were cross-linked with formaldehyde and incubated with sodium citrate to terminate the cross-linking reaction. Chromatin was sonicated with a sonication buffer followed by an incubated with 1 μg anti-Foxc1 or rabbit IgG overnight. Purification of the DNA fragments were carried out with Phenol-Chloroform extraction. After that, RT-qPCR were conducted for sample analysis. The primers are listed in **Table 7**.

**Table 7:** ChIP primer sequences.

Primer	Sequence (5′to 3′)	Product (bp)
Runx2-1-F	TTGTAAGAGCCGCCACGTAA	145
Runx2-1-R	AGCTCTGCTTGCAAACTCCT	
Runx2-2-F	CCTTACAGGAGTGTGGGCTC	103
Runx2-2-R	TAAGGCCTTCCTGGCATTCAG	
Runx2-3-F	AGGCAGTCCCACTTTACTTTG	74
Runx2-3-R	CAAGCACTATTACTGGAGAGACAGA	

### Animals and Experiment Design *In Vivo*

Three-month-old female Sprague Dawley rats with an average weight of 232 ± 7 g were enrolled. The experiment was reviewed and approved by the Ethics Committees of the Shanghai 9th People’s Hospital, School of Medicine, SJTU. A cranial defect model was established to determine the role of Foxc1 in bone regeneration induced by intermittent PTH treatment *in vivo*. After anesthetization of 10% chloral hydrate by intraperitoneal injection (4 ml per kg of body weight), a 1.0-cm sagittal cut was made through the skin on the scalp, and the periosteum was carefully elevated. When the skull was exposed, a 5-mm diameter bone defect was made in the midline by a 5-mm diameter round bur and cooled with continuous saline buffer irrigation. Scaffolds seeded with cells were then implanted into the defects followed by incision closure.

Thirty-two rats were randomly allocated into four groups: Group A, defect+β-TCP scaffolds+ BMSCs+ PBS (n = 8); Group B, defect+ β-TCP scaffolds+ BMSCs+ intermittent PTH treatment (n = 8); Group C, defect+ β-TCP scaffolds+ intermittent PTH treatment+ AAV-scramble BMSCs (n = 8); and Group D, defect+ β-TCP scaffolds+ AAV-shFoxc1 BMSCs + intermittent PTH treatment (n = 8). β-TCP scaffolds were from Shanghai Institute of Ceramics, Chinese Academy of Sciences. The scaffold diameter was 5 mm, and its thickness was 1.5 mm, while the pore size was around 350 to 500 μm, and the average porosity being about 80%. BMSCs of different groups were washed from the culture dishes. After centrifugation, BMSCs were resuspended in serum‐free DMEM medium. Next, BMSCs were seeded at a concentration of 1.0 × 10^5^ cells per ml on to the scaffolds and incubated for 4 h before implantation (Fan et al., 2016). Intermittent PTH treatment was achieved by intraperitoneally injected with rhPTH1–34 (80 μg/kg) (Kang et al., 2014) or PBS once a day for 8 weeks. Eight weeks later, micro-CT examination and bone parameter analysis were performed after fixation.

### Micro-Computed Tomography Scans

The samples were collected and fixed in 4% PFA for micro-computed tomography (μ-CT) examination (GE Healthcare Life Sciences, USA). Thickness of the slices was 0.01 mm. Three-dimensional reconstructions were performed, and parameters regarding bone mineral density were analyzed (GE Healthcare Life Sciences, USA).

### H&E Staining

The samples were collected and fixed in 4% PFA and further demineralized in 0.5 M ethylenediaminetetraacetic acid (EDTA). After that, the samples were embedded and cut with slices of 5-mm thick for regular H&E staining, and images were obtained with an upright metallurgical microscope (Nikon, Japan).

### Statistical Analysis

The experiments were all carried out for at least three times. All data were displayed as mean ± SD, and the statistical analysis was carried out by SPSS 20.0 software (SPSS Inc., USA). Differences between two groups were evaluated by two-tailed Student’s t test. ANOVA was used to determine significance between multiple groups. Levene’s test was firstly used for testing the assumption that the group variances are homogenous. When the homogeneity of variances assumption was met, the overall comparison was performed using the F test and Tukey test was utilized for the pairwise comparisons. When the homogeneity of variances assumption was not met, Welch’s test was used and Dunnett’s T3 test was utilized for the pairwise comparisons. *P* < 0.05 was considered statistically significant.

## Results

### Expression Level of Foxc1 Induced by Intermittent PTH Treatment

**Figure 1:**
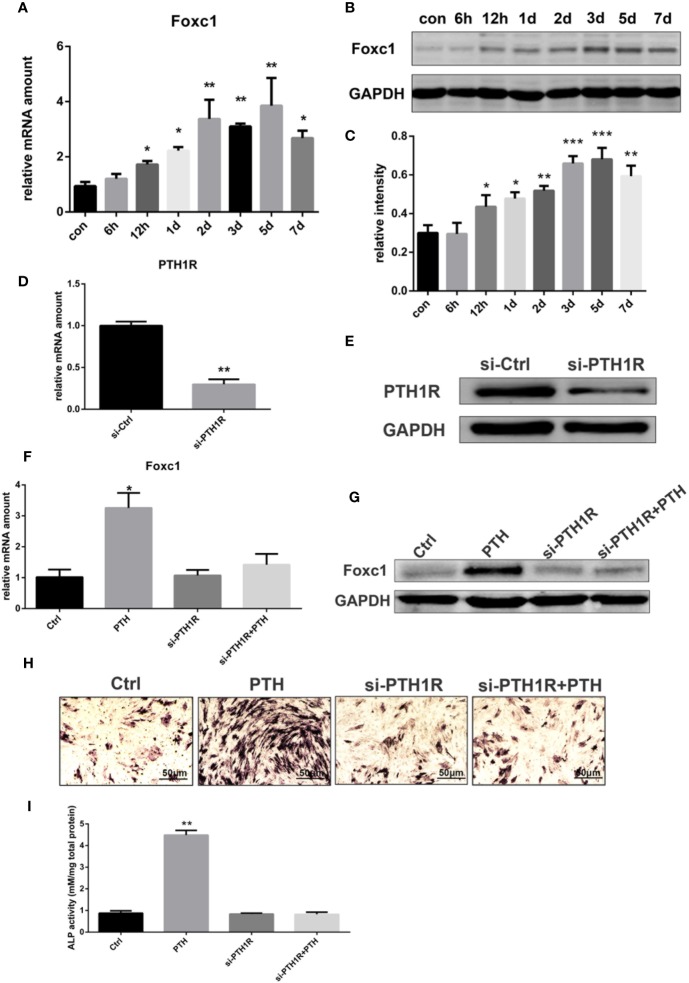
Expression of Foxc1 was increased by intermittent PTH treatment and PTH1R silencing inhibited the expression of Foxc1 and PTH-induced osteogenic differentiation. **(A)** mRNA expression level of Foxc1 at different time points induced by intermittent PTH treatment for 7 days in rBMSCs. **(B, C)** Protein expression level and quantitative analysis of Foxc1 at different time points by intermittent PTH treatment for 7 days in rBMSCs. **(D, E)** PTH1R mRNA and protein expression at 48 h after transfection in rBMSCs in control group (si-Ctrl) and PTH1R silencing group (si-PTH1R). **(F, G)** Foxc1 mRNA and protein expression on the 3rd day in control group (Ctrl), intermittent PTH treatment group (PTH), PTH1R silencing group (si-PTH1R) and PTH1R silencing group with intermittent PTH treatment (si-PTH1R+PTH). **(H, I)** ALP staining and its quantitative analysis on the 7th day in control group (Ctrl), intermittent PTH treatment group (PTH), PTH1R silencing group (si-PTH1R) and PTH1R silencing group with intermittent PTH treatment (si-PTH1R+PTH). Scale bar = 50 μm. *p < 0.05, **p < 0.01, ***p < 0.001.

To identify the effects of PTH-induced osteogenic differentiation on the expression level of Foxc1, rBMSCs were treated with intermittent PTH as indicated. The qPCR results indicated that mRNA expression level of Foxc1 increased gradually and PTH stimulated a significant increase to the maximal level on the 5th day (**Figure 1A**). Western blot exhibited consistent results with qPCR (**Figures 1B, C**), revealing that expression level of Foxc1 was upregulated during osteogenic induction by intermittent PTH treatment.

### Effect of PTH1R Silencing on the Expression of Foxc1 and PTH-Induced Osteogenic Differentiation

As parathyroid hormone 1 receptor (PTH1R) is the predominant receptor of PTH, the expression of PTH1R in rBMSCs was inhibited by RNAi-targeted silencing to further investigate the effect of PTH/PTH1R on Foxc1 expression level and on the role of osteogenic differentiation by PTH treatment. Forty-eight hours after transfection, the expression level of PTH1R in control group was relatively high, while that in si-PTH1R group expressed a remarkable decrease (**Figures 1D, E**). To determine the impacts of PTH1R silencing on Foxc1 expression during osteogenesis induced by intermittent PTH treatment, expression levels of Foxc1 were detected on the 3rd day. It turned out that the elevated Foxc1 expression level induced by intermittent PTH treatment was inhibited by PTH1R silencing, while rescued by PTH supplement after PTH1R silencing (**Figures 1F, G**). The results of ALP staining on the 7th day showed similar tendency with the expression of Foxc1, indicating the potential role of Foxc1 on osteogenic differentiation induced by intermittent PTH treatment (**Figures 1H, I**).

### Effect of Foxc1 on Osteogenic Differentiation Induced by Intermittent PTH Treatment *In Vitro*

**Figure 2:**
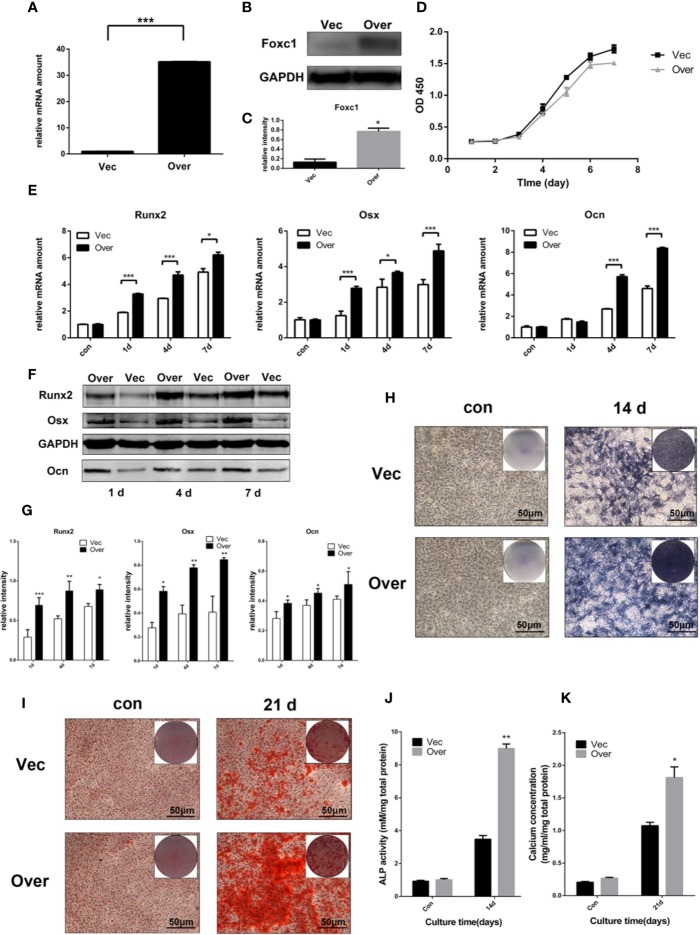
Foxc1 overexpression stimulated osteogenic differentiation induced by intermittent PTH treatment *in vitro*. **(A)** mRNA expression level of Foxc1 in Lv-vector and Lv-Foxc1 MC3T3-E1 cells at 48 h after transfection. **(B, C)** Protein expression level and quantitative analysis of Foxc1 at 48 h after transfection. **(D)** Cell proliferation rate in Lv-vector and Lv-Foxc1 MC3T3-E1 cells. **(E)** mRNA expression levels of the osteogenic markers, Runx2, Osx and Ocn on the 1st day, 4th day, and 7th day after exposed to intermittent PTH treatment. **(F, G)** Protein expression level and quantitative analysis of the osteogenic markers, Runx2, Osx, and Ocn on the 1st day, 4th day, and 7th day after exposed to intermittent PTH treatment. **(H, J)** ALP staining and activity in Lv-Foxc1 and Lv-vector MC3T3-E1 cells after 2 weeks of intermittent PTH treatment. **(I, K)** Alizarin red S staining and calcium concentration in Lv-Foxc1 and Lv-vector MC3T3-E1 cells after 3 weeks of intermittent PTH treatment. **(I, K)**. Scale bar = 50 μm. *p < 0.05, **p < 0.01, ***p < 0.001.

In order to evaluate whether Foxc1 takes a major part in PTH-induced osteogenesis, gain- and loss-of-function assays with Foxc1 overexpressing and Foxc1-knockdown cells were performed. qPCR results revealed that Lv-Foxc1 MC3T3-E1 cells showed a strong mRNA expression of Foxc1 (**Figure 2A**) and a correspondingly high protein production compared with Lv-vector MC3T3-E1 (**Figures 2B, C**), indicating that lentivirus was successfully transfected, and overexpression of Foxc1 in MC3T3-E1 cells was stably induced. Meanwhile, cell proliferation rate measured by CCK8 indicated that Lv-Foxc1 did not have significant impact on proliferation rate of MC3T3-E1 cells (**Figure 2D**). Compared with Lv-vector MC3T3-E1 cells, Runx2, and Osx mRNA expression levels were higher in Lv-Foxc1 MC3T3-E1 cells on the 1st day, 4th day, and 7th day after exposed to intermittent PTH treatment. The mRNA expression level of Ocn was significantly upregulated on the 4th day and 7th day (**Figure 2E**). In the meantime, overexpression of Foxc1 resulted in an increase in Runx2, Osx, and Ocn protein expression levels on the 1st day, 4th day, and 7th day (**Figures 2F, G**). ALP expression was well detected in Lv-Foxc1 MC3T3-E1 cells after 14 days of intermittent PTH treatment (**Figure 2H**), and the ALP activity increased by nearly three folds compared with Lv-vector MC3T3-E1 cells (**Figure 2J**). Similarly, Alizarin red S staining and calcium concentration assay both exhibited higher osteogenic potential in Lv-Foxc1 MC3T3-E1 cells (**Figures 2I, K**).

**Figure 3:**
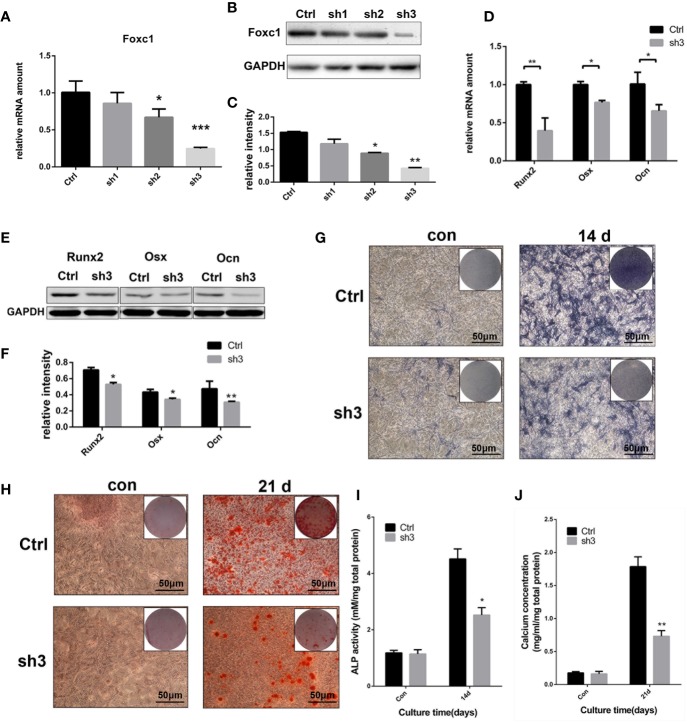
Foxc1 knockdown inhibited osteogenic differentiation induced by intermittent PTH treatment *in vitro*. **(A)** mRNA expression level of Foxc1 in Lv-scramble, Lv-shFoxc1–1, Lv-shFoxc1–2, and Lv-shFoxc1–3 MC3T3-E1 cells at 48 h after transfection. **(B, C)** Protein expression level and quantitative analysis of Foxc1 at 48 h after transfection. **(D)** mRNA expression levels of the osteogenic markers, Runx2, Osx, and Ocn on the 1st day, 4th day, and 7th day after exposed to intermittent PTH treatment in Lv-scramble and Lv-shFoxc1–3 MC3T3-E1 cells. **(E, F)** Protein expression level and quantitative analysis of the osteogenic markers, Runx2, Osx, and Ocn on the 1st day, 4th day, and 7th day after exposed to intermittent PTH treatment in Lv-scramble and Lv-shFoxc1–3 MC3T3-E1 cells. **(G, I)** ALP staining and activity in Lv-scramble and Lv-shFoxc1–3 MC3T3-E1 cells after 2 weeks of intermittent PTH treatment. **(H, J)** Alizarin red S staining and calcium concentration in Lv-scramble and Lv-shFoxc1–3 MC3T3-E1 cells after 3 weeks of intermittent PTH treatment. Scale bar = 50 μm. *p < 0.05, **p < 0.01, ***p < 0.001.

At the same time, three shRNAs were designed to knock down the expression of Foxc1, and the efficiency was analyzed at protein and mRNA levels. qPCR and western blot results indicated that LV‐shFoxc1–2 and LV‐shFoxc1–3 could downregulate Foxc1 expression, and the inhibition effect of LV‐shFoxc1–3 was more obvious (**Figures 3A–C**). Expression levels of Runx2, Osx, and Ocn were significantly decreased after Foxc1 knocking down on the 4th day in the osteogenic differentiation process induced by intermittent PTH treatment (**Figures 3D–F**). ALP staining was performed 14 days after PTH-induced osteogenic differentiation. It turned out that LV‐shFoxc1–3 inhibited ALP activity on the 14th day (**Figure 3G**), while quantitative measurements confirmed the above results (**Figure 3I**). In addition, LV‐shFoxc1–3 MC3T3-E1 cells showed lighter alizarin red staining and less calcium nodule formation on the 21st day (**Figures 3H, J**). So far, our gain- and loss-of-function experiment results demonstrated that Foxc1 can promote osteogenic differentiation induced by intermittent PTH treatment *in vitro*.

### Role of Foxc1 in Regulating Runx2 Promoter Activity and Transcription

**Figure 4:**
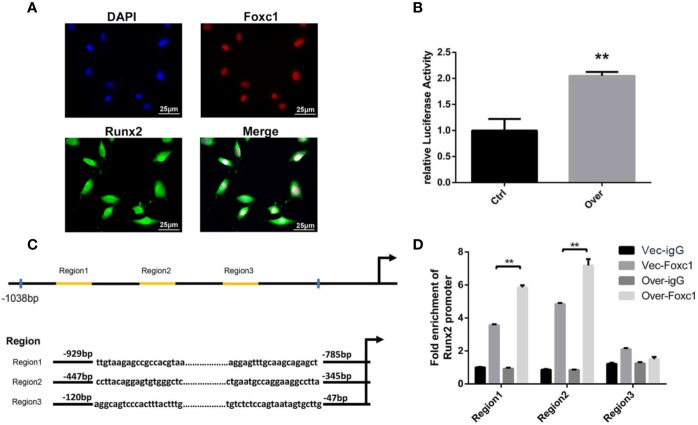
Foxc1 directly regulated transcriptional activity of Runx2 by binding to its promoter. **(A)** Expression of Foxc1 and Runx2 in MC3T3-E1 cells by immunofluorescence analysis. **(B)** Luciferase reporter assay of Runx2 promoter activity. **(C)** Putative Foxc1-binding sites in the proximal promoter region of the mice Runx2 gene. **(D)** Chromatin immunoprecipitate obtained in the ChIP assay quantified by qPCR for detecting the binding of Foxc1 to the Runx2 gene promoter region. Scale bar = 25 μm. **p < 0.01.

As the immunofluorescence analysis indicated the nuclear co-localization of Foxc1 with Runx2 (**Figure 4A**), we further performed a luciferase reporter assay for evaluation of Runx2 promoter activity in PTH-induced osteogenic differentiation to investigate the potential role of Foxc1 in modulating Runx2 gene promoter activity. We found that the promoter activity of Runx2 was significantly upregulated by Foxc1 overexpression (**Figure 4B**). These results indicated that Foxc1 could enhance the transcriptional activity of the Runx2 promoter.

Furthermore, in order to identify the underlying regulation mechanism of Foxc1 on Runx2 expression, the structure and function of mouse Runx2 gene promoter were examined. Bioinformatics analysis through the JASPAR database revealed three potential Foxc1-binding elements in mice Runx2 promoter region (**Figure 4C**). The binding of Foxc1 to the promoter region of Runx2 was further detected through a chromatin immunoprecipitation assay using anti-Foxc1 as described. Chromatin immune complexes obtained from PTH-induced osteoinductive medium of MC3T3-E1 cells upon 3 days were analyzed by qPCR using Runx2 promoter gene specific primers for the Foxc1-binding region. As shown in **Figure 4D**, functional Region1 and Region2, located at 929 to −785 bp, and −447 to −345 bp relative to transcription initiation site, were responsive to the stimulation from Foxc1.

### Effect of Foxc1 in the Process of Bone Regeneration Induced by Intermittent PTH Treatment *In Vivo*

**Figure 5:**
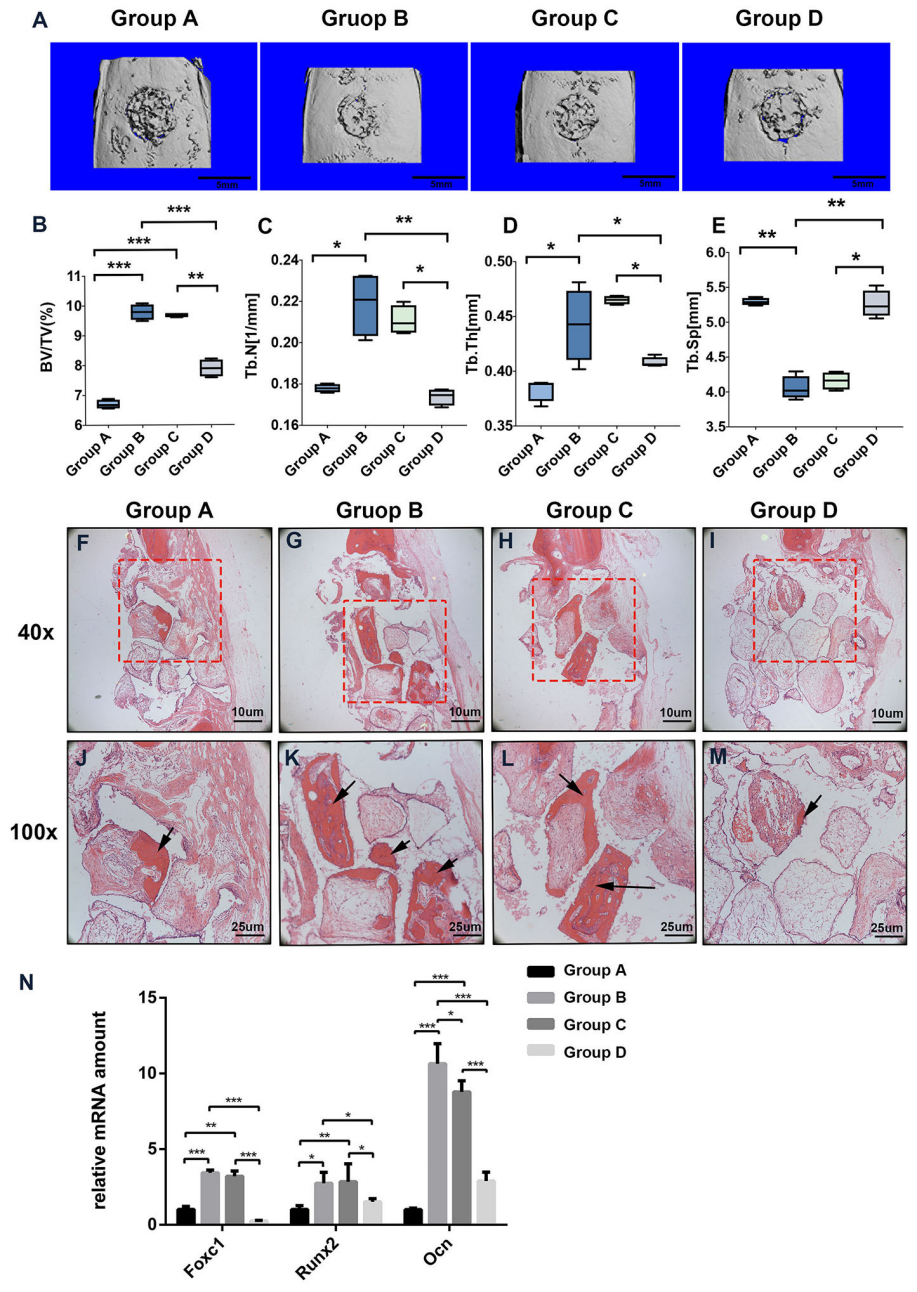
Foxc1 stimulates osteogenic differentiation induced by intermittent PTH treatment *in vivo*. **(A)** Three-dimensional reconstruction images of the defect sites by micro-CT in different groups at 8 weeks. **(B–E)** BV percentage **(B)**, trabecular number **(C)**, trabecular thickness **(D)**, and the number of trabecular **(E)** in different groups were quantified. **(F–M)** H&E staining of the defect sites in different groups at 8 weeks. **(N)** mRNA expression levels of Foxc1, Runx2, and Ocn in different groups at 8 weeks. Scale bar = 5 mm in **(A)**, Scale bar = 10 μm in **(F–I)**, Scale bar = 25 μm in **(J–M)**. **(J–M)** are local amplification of boxed area in **(F–I)**, respectively. Arrow indicates new bone formation. *p < 0.05, **p < 0.01, ***p < 0.001.

To evaluate whether PTH modulates Foxc1 function *in vivo*, we utilized a rat cranial defect model and implanted β-TCP scaffolds loaded with BMSCs. After 8 weeks of implantation, 3D reconstruction images of μ-CT for the defect area (**Figure 5A**) showed little mineralization in PBS+BMSCs control group (group A) and only a slight amount of mineralization in PTH+ AAV-shFoxc1 BMSCs group (group D). On the other hand, newly formed bone was detected at the edge of the defect area in both PTH + BMSCs group (group B) and PTH+ AAV-scramble BMSCs group (group C). The diameters of the defect area decreased more or less in all the groups. New bone formation in groups B and C was more obvious compared with groups A and D, and that in group B was the most prominent one. Bone volume density (BV/TV) analysis indicated that bone volume of group B was significantly higher than that of groups A and D, while no difference was detected significantly within groups C and B. Meanwhile, bone volume of group C was also higher than that of groups A and D (**Figure 5B**). Trabecular number of group B was greatly upregulated compared with that of groups A and D, and so was that of group C, while no difference was detected significantly within groups B and C neither (**Figure 5C**). The trabecular thickness (Tb.Th) exhibited same tendency with the number of trabecular (**Figure 5D**), and results of trabecular bone spacing (Tb.Sp) also displayed similar patterns, indicating more newly formed bone in groups B and C than in groups A and D (**Figure 5E**).

Consistent with the μ-CT results, instead of new bone, more fibrous connective tissues were detected in the defect area of groups A and D. In contrast, more newly formed bone and osteoid appeared in the bone stump in groups B and C, particularly in group B (**Figures 5F–M**). Meanwhile, the mRNA expression levels of Foxc1, Runx2 and Ocn in groups B and C were also greatly upregulated compared with those in groups A and D (**Figure 5N**). These results indicated that β-TCP scaffolds loaded with BMSCs and induced by intermittent PTH treatment at the same time have the best bone regeneration potential among all the groups, while knocking down of Foxc1 could inhibit the bone regeneration process induced by intermittent PTH treatment, suggesting the vital role of Foxc1 in bone regeneration induced by intermittent PTH treatment *in vivo*.

## Discussion

Intermittent PTH administration has been used for clinical bone stimulation in osteoporosis patients for decades (Vrahnas et al., 2016). As a common receptor of PTH, PTH1R is in charge of various cellular processes and generally expressed in cells and tissues. In the skeleton, the expression of PTH1R could be detected in osteocytes and osteoblasts, with higher levels in the former cells (Delgado-Calle et al., 2017). However, the mechanism of which PTH/PTH1R stimulates skeletal formation still needs exploration. Herein, we revealed that elevated expression of Foxc1 and upregulated ALP activity induced by intermittent PTH treatment was inhibited by PTH1R silencing, while rescued by PTH supplement, indicating the potential role of Foxc1 on osteogenic differentiation in response of intermittent PTH treatment targeting PTH1R.

Previous researches have shown that Foxc1 is an important regulator in both endochondral ossification and intramembranous ossification. Foxc1-mutant mice die during embryonic period due to similar defects in skeleton, oculus, and cardiovascular system, along with hydrocephalus and hypoplasia of cerebellum (Seo and Kume, 2006). It is worth noting that the most severe skeletal mutations of Foxc1 knockout mice is calvarium deficiency (Rice et al., 2003), and thus we utilized a rat cranial defect model in this study to evaluate whether PTH modulates Foxc1 function *in vivo*. Meanwhile, mutations in Foxc1 in humans are often related with autosomal dominant diseases as the Axenfeld-Rieger and Dandy Walker syndromes. Malformations of the Axenfeld-Rieger syndrome includes tooth abnormalities, maxillary hypoplasia, dysplasia of the anterior eye segment, and defects of the cardiac flow tract (Seifi and Walter, 2018). As the most common congenital disorders of cerebellum, affected individuals of Dandy-Walker syndrome were often detected with duplications or deletions within the Foxc1 gene(Haldipur et al., 2017). What is more, Foxc1 is profoundly expressed in pre-osteogenic stem cells and is crucial for the formation of cell niche (Omatsu et al., 2014). Also, in self-renewing stem cells, Foxc1 activates the BMP and Nfatc1 pathways and reinforces the quiescence (Wang et al., 2016b). Notably, recent studies showed that ossification centers of long bones were lessened in Foxc1 knockout mice and that Foxc1 could regulate the expression of PTHrP, indicating functional links between Foxc1- and PTH-related osteogenic differentiation (Hopkins et al., 2016a). As the effect of Foxc1 on osteogenesis in response of intermittent PTH has not been verified yet, we used intermittent PTH in this study to induce osteogenic differentiation. After confirming the upregulated expression level of Foxc1, we then revealed that Foxc1 positively regulate osteogenic differentiation in response of PTH *in vitro* using gain- and loss-of-function approaches in MC3T3-E1 cells. At the same time, a rat cranial defect model and the β-TCP scaffolds loaded with BMSCs or AAV-shFoxc1 BMSCs or AAV-scramble BMSCs with/without PTH treatment further confirmed that Foxc1 could also promote bone regeneration *in vivo* induced by PTH.

Furthermore, as a transcription factor, it has been reported that Foxc1 could directly bind on the enhancer region of Bmp and inhibit phosphorylation of Smad1/5/8, playing an essential part in Bmp-dependent Msx2 activation (Sun et al., 2013b). Foxc1 was also reported to be directly regulated by the Yap-Tead complex, suggesting its essential role in diversification and development of central nerve crests (Wang et al., 2016a). Thus, more target genes of Foxc1 should be identified in bone-forming cells for better understanding of osteogenesis. Notably, we found that the expression level of Runx2, the key transcription factor for osteogenesis, changed with Foxc1 and immunofluorescence analysis also indicated the nuclear co-localization of Foxc1 with Runx2. In our research, we revealed that the upregulation of Foxc1 expression appeared at an early stage in osteogenic differentiation *in vitro*. Researchers also found that Foxc1 was detected in ectoderm and craniofacial mesenchyme of mouse embryos at E8.5 and in the first zygomatic arch at E9.5, while Runx2 was detected in the osteogenic precursor matrix at E10.5, suggesting the potential regulating effect of Foxc1 on Runx2 in osteogenic differentiation (Sun et al., 2013a; Mya et al., 2018b). Such hypothesis was confirmed by luciferase reporter assay, and it turned out that Foxc1 could enhance the transcriptional activity of Runx2.

After that, we analyzed the structure and function of mouse Runx2 gene promoter in order to elucidate the underlying regulation mechanism of Foxc1 on Runx2 expression. It was reported that Runx2 had two isoforms with different N-terminals and was transcribed from the distal and proximal promoters, named as P1 and P2. The P2 promoter maintains basal expression level of Runx2 in thymus, cartilage, periosteum, and cranial suture (Ng et al., 2007), while the P1 or the “bone-related” promoter is widely involved in bone development and plays a predominant role in hypertrophic differentiation of chondrocytes and osteoblasts (Ducy et al., 1997). For osteoblastic-related cells, it is reported that Runx2 was mostly transcribed from the P1 promoter. Further studies demonstrated that Sp1/Elk1, Tcf7/Ctnnb1, Fosb/Jund, Msx2, Dlx5, and Hoxa10 bind to the P1 promoter of Runx2 in the proximal 700-bp region and thus regulate its transcriptional activity *in vitro* (Komori, 2017; Komori, 2018). Based on the former data and bioinformatics analysis through the JASPAR database, we herein used a luciferase reporter assay and a ChIP assay to verify the regulation effect of Foxc1 on Runx2. Our results revealed that Foxc1 not only enhanced the promoter activity of Runx2, but also bound to the two promoter regions of Runx2, Region1, and Region2, with Region2 located in the P1 promoter region. Therefore, it was confirmed that Foxc1 could bind to the promoter region and directly regulate Runx2.

It is worth noting that Berry *et al*. reported that Foxc1 is a short-lived protein with its protein levels and activity regulated by post-translational modifications, and serine 272 is an important residue in maintaining proper stability of Foxc1(Berry et al., 2006). Interestingly, Ito *et al*. pointed out that inhibiting phosphorylation of serine 272 residue impairs the stability, but increases the transcriptional activity of Focx1 (Ito et al., 2014). Meanwhile, Hayashi *et al*. found that Foxc1 have 10 potential phosphorylation sites for ERK but there is no potential ERK1/2 phosphorylation site in the DNA-binding domains (Hayashi and Kume, 2008). These indicated that the stability, transcriptional activity, and protein level of Foxc1 are regulated by various complicated factors, and further research should be carried out for obtaining more information.

**Figure 6:**
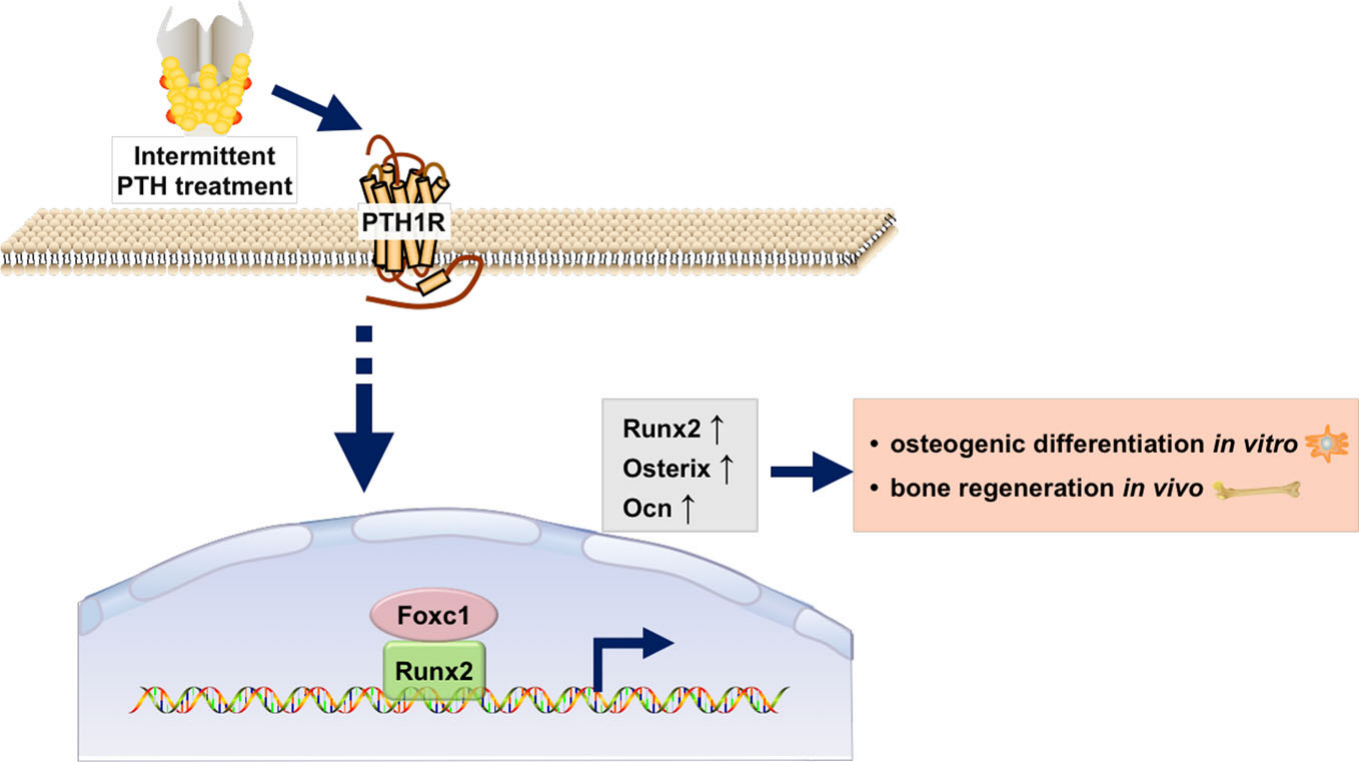
Schematic illustration of the regulation of Runx2 by Foxc1 in response of intermittent PTH.

In conclusion, as was shown in **Figure 6**, our study revealed that the expression of Foxc1 was elevated in intermittent PTH treatment and Foxc1 could promote *in vitro* osteogenic differentiation and *in vivo* bone regeneration induced by intermittent PTH. Further exploration of the molecular regulatory mechanism revealed that Foxc1 could bind to the P1 promoter of Runx2 directly, which played an indispensable role in osteogenesis and bone mineralization. These results help us understand the role of Foxc1 in osteogenesis in response of intermittent PTH and may provide a possible molecular target for bone regeneration.

## Data Availability Statement

The datasets generated for this study are available on request to the corresponding authors.

## Ethics Statement

The animal study was reviewed and approved by the Animal Ethical Committee at the Ninth People’s Hospital affiliated to Shanghai Jiaotong University, School of Medicine.

## Author Contributions

The conception and design of the study: GS and JS. Project administration, investigation, and drafting the article: NO and HL. Data curation and formal analysis: NO, MW, and HS. All authors read and approved the final manuscript.

## Funding

This work was supported by grants from National Nature Science Foundation of China (81771036, 81600827, 81570947), and Shanghai international science and technology cooperation projects (17410710500).

## Conflict of Interest

The authors declare that the research was conducted in the absence of any commercial or financial relationships that could be construed as a potential conflict of interest.
