# Supplementary material for: The Transcription Factor Foxc1 Promotes Osteogenesis by Directly Regulating Runx2 in Response of Intermittent Parathyroid Hormone (1–34) Treatment
Source: Front Pharmacol. 2020 May 5;11:592. doi: 10.3389/fphar.2020.00592 (PMC7216818; doi:10.3389/fphar.2020.00592)
Supplement: Supplementary file 1 [file DataSheet_1.docx]

**Supplementary data**

**1. Sequencing results for the expression vector of mouse Foxc1**

| **Sequencing results:** | **m-Foxc1 overexpression** |
| --- | --- |
| GAATTCatgcaggcgcgctactcggtgtccagccccaactccctgggagtggtgccctacctcggcggcgagcagagctactatcgcgctgccgcggcggcggccgggggcggctacaccgccatgccggcccctatgagcgtgtactcgcaccctgctcacgccgagcagtacccgggcagcatggcgcgcgcctacgggccttatacgccgcagccgcagcccaaggacatggtgaagccgccctacagctacatcgctcttatcaccatggccatccagaatgccccggacaagaagatcactctgaatggcatctaccagttcatcatggaccgcttccccttctatcgggacaataagcagggctggcagaacagcatacggcacaacctctcgctcaacgagtgcttcgtcaaggtgccccgcgacgacaagaagccaggcaagggcagctactggacgctcgacccggactcctacaacatgttcgagaacggcagcttcctgcggcggcggcggcgcttcaagaagaaggacgcagtgaaggacaaggaggagaagggccggctgcacctccaagaaccgccaccgccgcaggccggccgccagcccgcgcccgcgcccccggagcaggccgagggctccgctcccgggccacagccgccgcccgtgcgcatccaggacatcaagacggagaacggtacgtgtccctcgcctccccagcccctgtccccggctgccgccctaggcagcggcagcgccgccacagtgcccaaaatcgagagccccgacagcagcagcagcagcttgtcgagcgggagcagccccccgggcagcctgccgtcggcgcggccgctcagcctggacgctgcagaacccgcgccgccgccacagcctgcgccgccgccgcatcacagccagggcttcagcgtggacaacatcatgacgtcgctgcgggggtcgccgcagggttcggccgctgagctcggttccggcctcctggcctcggcggctgcgtcctcgcgcgcgggcatcgcgcccccgctggcgctgggtgcctactctccgggccagagctccctctacagctccccctgcagccagagctccagtgcgggcagttcgggcggcgggggtggcggcggcggcggaggcggcggcagcagcagcgctgcgggtacggggggcgccgccacttaccactgcaacctgcaggctatgagcctgtacgcggcgggcgagcgtggcggccacttgcagggtccggcgggaggcgcgggcagcgcggcggtggacgaccccctgcccgactactcgctgcctccagcgaccagcagcagctcttcgtccctgagtcatggcgggggcggccaggaggccagccaccaccctgcatcccaccagggccgactcacctcgtggtacctgaaccaggcaggtggagacctgggccacttggcgagcgcggcggcggctgcggcggccgcaggctaccctggccagcagcagaacttccactcggtgcgggaaatgttcgagtctcagcggatcggcttgaacaactccccggtgaatgggaatagtagctgtcagatggctttccctgccagtcagtctctgtaccgcacgtcgggggctttcgtctatgactgtagcaaattctgaGGATCC | |

**2. Sequencing results for interfering plasmids of mouse Foxc1**

| **Sequencing results:** | **m-shFoxc1-1** |
| --- | --- |
| TGCATACGATACAGGCTGTTAGAGAGATAATTAGAATTAATTTGACTGTAAACACAAAGATATTAGTACAAAATACGTGACGTAGAAAGTAATAATTTCTTGGGTAGTTTGCAGTTTTAAAATTATGTTTTAAAATGGACTATCATATGCTTACCGTAACTTGAAAGTATTTCGATTTCTTGGCTTTATATATCTTGTGGAAAGGACGAGGATCCGGAATAGTAGCTGTCAGATGGTTCAAGAGACCATCTGACAGCTACTATTCCTTTTTTGAATTCTAGTTATTAATAGTAATCAATTACGGGGTCATTAGTTCATAGCCCATATATGGAGTTCCGCGTTACATAACTTACGGTAAATGGCCCGCCTGGCTGACCGCCCAACGACCCCCGCCCATTGACGTCAATAATGACGTATGTTCCCATAGTAACGCCAATAGGGACTTTCCATTGACGTCAATGGGTGGAGTATTTACGGTAAACTGCCCACTTGGCAGTACATCAAGTGTATCATATGCCAAGTACGCCCCCTATTGACGTCAATGACGGTAAATGGCCCGCCTGGCATTATGCCCAGTACATGACCTTATGGGACTTTCCTACTTGGCAGTACATCTACGTATTAGTCATCGCTATTACCATGGTGATGCGGTTTTGGCAGTACATCAATGGGCGTGGATAGCGGTTTGACTCACGGGGATTTCCAAGTCTCCACCCCATTGACGTCAATGGGAGTTTGTTTTGGCACCAAAATCAACGGGACTTTCCAAAATGTCGTAACAACTCCGCCCCATTGACGCAAATGGGCGGTAGGCGTGTACGGTGGGAGGTCTATATAAGCAGAGCTGGTTTAGTGAACCGTCAGATCCGCTAGCGCTACCGGTCGCCACCATGGCCCAGTCCAAGCACGGCCTGACCAAGGAGATGACCATGAAGTACCGCATGGAGGGCTGCGTGGACGGCCACAAGTTCGTGATCACCGGCGAGGGCATCGGCTACCCCTTCAAGGGCAAGCAGGCCATCAACCTGTGCGTGGTGGAGGGCGGCCCCTTGCCCTTCGCC | |
| **Sequencing results:** | **m-shFoxc1-2** |
| TGCCATACGATACAGGCTGTTAGAGAGATAATTAGAATTAATTTGACTGTAAACACAAAGATATTAGTACAAAATACGTGACGTAGAAAGTAATAATTTCTTGGGTAGTTTGCAGTTTTAAAATTATGTTTTAAAATGGACTATCATATGCTTACCGTAACTTGAAAGTATTTCGATTTCTTGGCTTTATATATCTTGTGGAAAGGACGAGGATCCGCTTCAGCGTGGACAACATCATTCAAGAGATGATGTTGTCCACGCTGAAGCTTTTTTGAATTCTAGTTATTAATAGTAATCAATTACGGGGTCATTAGTTCATAGCCCATATATGGAGTTCCGCGTTACATAACTTACGGTAAATGGCCCGCCTGGCTGACCGCCCAACGACCCCCGCCCATTGACGTCAATAATGACGTATGTTCCCATAGTAACGCCAATAGGGACTTTCCATTGACGTCAATGGGTGGAGTATTTACGGTAAACTGCCCACTTGGCAGTACATCAAGTGTATCATATGCCAAGTACGCCCCCTATTGACGTCAATGACGGTAAATGGCCCGCCTGGCATTATGCCCAGTACATGACCTTATGGGACTTTCCTACTTGGCAGTACATCTACGTATTAGTCATCGCTATTACCATGGTGATGCGGTTTTGGCAGTACATCAATGGGCGTGGATAGCGGTTTGACTCACGGGGATTTCCAAGTCTCCACCCCATTGACGTCAATGGGAGTTTGTTTTGGCACCAAAATCAACGGGACTTTCCAAAATGTCGTAACAACTCCGCCCCATTGACGCAAATGGGCGGTAGGCGTGTACGGTGGGAGGTCTATATAAGCAGAGCTGGTTTAGTGAACCGTCAGATCCGCTAGCGCTACCGGTCGCCACCATGGCCCAGTCCAAGCACGGCCTGACCAAGGAGATGACCATGAAGTACCGCATGGAGGGCTGCGTGACGGCCACAAGTTCGTGATCACCGGCGAGGGCATCGGCTACCCCTTCAAGGGCAAGCAGGCCATCAACCTGTGCGTGGTGGA | |
| **Sequencing results:** | **m-shFoxc1-3** |
| TGCATACGATACAGGCTGTTAGAGAGATAATTAGAATTAATTTGACTGTAAACACAAAGATATTAGTACAAAATACGTGACGTAGAAAGTAATAATTTCTTGGGTAGTTTGCAGTTTTAAAATTATGTTTTAAAATGGACTATCATATGCTTACCGTAACTTGAAAGTATTTCGATTTCTTGGCTTTATATATCTTGTGGAAAGGACGAGGATCCGTAGCAAATTCTGACCCTATTCCTCGAGGAATAGGGTCAGAATTTGCTATTTTTTAATTCTAGTTATTAATAGTAATCAATTACGGGGTCATTAGTTCATAGCCCATATATGGAGTTCCGCGTTACATAACTTACGGTAAATGGCCCGCCTGGCTGACCGCCCAACGACCCCCGCCCATTGACGTCAATAATGACGTATGTTCCCATAGTAACGCCAATAGGGACTTTCCATTGACGTCAATGGGTGGAGTATTTACGGTAAACTGCCCACTTGGCAGTACATCAAGTGTATCATATGCCAAGTACGCCCCCTATTGACGTCAATGACGGTAAATGGCCCGCCTGGCATTATGCCCAGTACATGACCTTATGGGACTTTCCTACTTGGCAGTACATCTACGTATTAGTCATCGCTATTACCATGGTGATGCGGTTTTGGCAGTACATCAATGGGCGTGGATAGCGGTTTGACTCACGGGGATTTCCAAGTCTCCACCCCATTGACGTCAATGGGAGTTTGTTTTGGCACCAAAATCAACGGGACTTTCCAAAATGTCGTAACAACTCCGCCCCATTGACGCAAATGGGCGGTAGGCGTGTACGGTGGGAGGTCTATATAAGCAGAGCTGGTTTAGTGAACCGTCAGATCCGCTAGCGCTACCGGTCGCCACCATGGCCCAGTCCAAGCACGGCCTGACCAAGGAGATGACCATGAAGTACCGCATGGAGGGCTGCGTGGACGGCCACAAGTTCGTGATCACCGGCGAGGGCATCGGCTACCCCTTCAAGGGCAAGCAGGCCATCAACCTGTGCGTGGTGAGGGCGGCCCCTTGCCCTTCGCCGAGGACATCTTTGTC | |

**3. Sequencing results for interfering plasmids of rat Foxc1**

| **Sequencing results:** | **r-shFoxc1** |
| --- | --- |
| GGAAGGGGGCTTTTTGCTTGCCTCCAGGCGATTTCTTGGGTTATATATCTTGTGGAAGGACGCGGGATCCGGGCACAACCTCTCGCTTAATGATCAAGAGTCATTAAGCGAGAGGTTGTGCTTTTTTAGATCTAAGCTTGCGGCCGCAGGAACCCCTAGTGATGGAGTTGGCCACTCCCTCTCTGCGCG | |
